# Determination of Levels of Organochlorine, Organophosphorus, and Pyrethroid Pesticide Residues in Vegetables from Markets in Dar es Salaam by GC-MS

**DOI:** 10.1155/2017/4676724

**Published:** 2017-02-09

**Authors:** John A. M. Mahugija, Farhat A. Khamis, Esther H. J. Lugwisha

**Affiliations:** ^1^Chemistry Department, University of Dar es Salaam, P.O. Box 35061, Dar es Salaam, Tanzania; ^2^Chief Government Chemist Laboratory Agency, Zanzibar, Tanzania

## Abstract

This study investigated the levels of pesticides and metabolites in vegetables from major markets in Dar es Salaam city, Tanzania. Samples of fresh cabbage, spinach, and onions from the markets were analysed for pesticide residues. Extraction was performed using acetone followed by dichloromethane : cyclohexane mixture and the extracts were cleaned up using Florisil. The compounds were determined by gas chromatography-mass spectrometry (GC-MS). Pesticides and metabolites were detected in 72.2% of the samples. The detected pesticide residues and their highest mean concentrations were* p,p*′-DDT 4.00 × 10^−3^ mg/kg,* p,p*′-DDD 6.40 × 10^−1^ mg/kg,* o,p*′-DDD 1.00 × 10^−2^ mg/kg, *α*-endosulfan 6.00 × 10^−1^ mg/kg, *β*-endosulfan 2.10 × 10^−1^ mg/kg, chlorpyrifos 3.00 mg/kg, and cypermethrin 4.00 × 10^−2^ mg/kg. The most frequently detected compounds were* p,p*′-DDD and chlorpyrifos. The order of contamination was spinach > cabbage > onions. Generally, there were no significant variations in concentrations of pesticide residues among samples and sampling sites, which indicated similarities in contamination patterns. The concentrations of contaminants were above the maximum residue limits (MRLs) in 33.3–50% of the samples. The findings indicated risks and concerns for public health.

## 1. Introduction

Pesticides are widely used in vegetables to control pests and diseases during farming, transportation, and storage. Pesticides are known to be the most important tool for the production of adequate food supply for an increasing world population and for the control of vector-borne diseases [1]. However, pesticides have some toxicological and environmental consequences, which include toxic residues in food substances and adverse effects on nontarget organisms. The gross and improper use of synthetic pesticides is a matter of much concern. Pesticides have been associated with a wide variety of human health hazards, ranging from acute impacts such as headache, vomiting, and diarrhoea to chronic impacts like cancer, reproductive harm, and endocrine disruption. Many people die from pesticides poisoning and other people suffer from various health effects [2, 3].

Vegetables are among the most frequently consumed food types in Tanzania. As vegetables are eaten either fresh or semiprocessed and due to improper agricultural practices of some farmers such as not observing the withholding periods after spraying, it could be expected that they contain high pesticide residues. Dar es Salaam is a big city hosting major transportation and commercial networks, markets, and industrial activities. The largest and busiest markets in Tanzania are found in Dar es Salaam. These markets are well known for their massive sales of vegetables that come from different areas of the country where pesticides are widely used. Thus, assessment of pesticides in samples from these markets could reflect the contamination status in the area and other areas. Literature surveys indicated that no study had been conducted on pesticide residues in vegetables in Tanzanian markets. Therefore, the above observations prompted the inception of this study.

## 2. Materials and Methods

### 2.1. Study Area and Sampling

**Figure 1:**
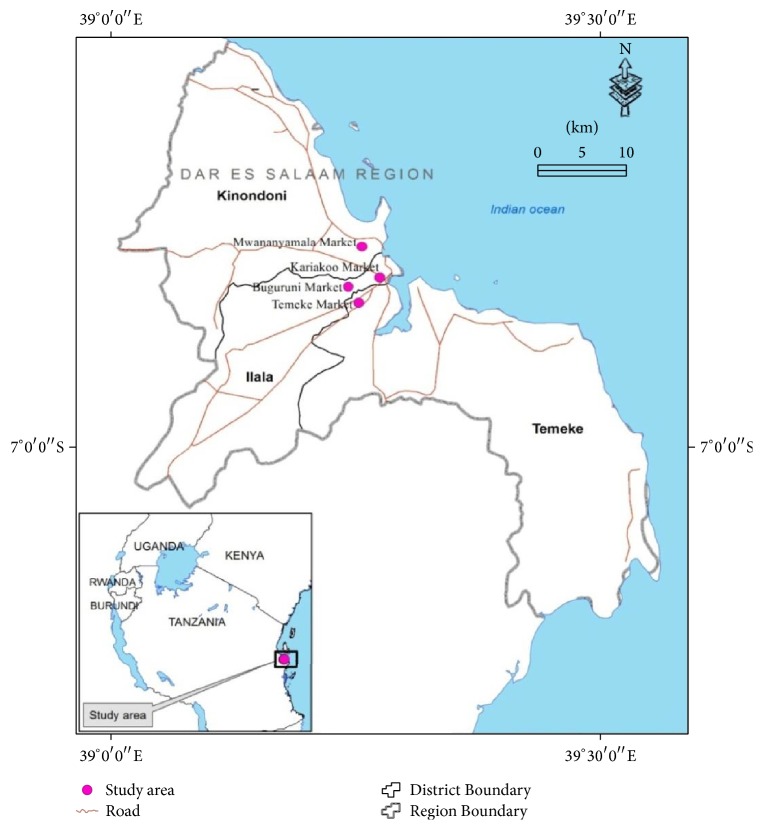
Map showing sampling sites.

Four major markets of Dar es Salaam city were randomly selected for collection of vegetables in December 2013. The samples were collected from Kariakoo (Kko), Buguruni (Bgn), Mwananyamala (Mny), and Temeke (Tmk) markets in Dar es Salaam region. All these markets are common selling places for vegetables and the vegetables come from different regions of Tanzania.  Figure 1 is a map showing the locations of the sampling sites.

The selected vegetables collected were* Brassica oleracea* var.* capitata* (cabbage),* Spinacia oleracea* (spinach), and* Allium cepa* (onions). Sampling was conducted by applying standard guidelines [4]. A total of 72 samples (200–500 g of each sample) were purchased from the markets, separately wrapped in aluminium foil, and placed into polythene bags. In the laboratory, the samples were stored in a freezer.

### 2.2. Sample Preparation and Processing

Sample preparation and extraction were conducted within 24 hours after sampling. Each sample was homogenized by using motor and pestle. The homogenized sample (20 g) was extracted with acetone (20 mL) and then with a mixture of dichloromethane : cyclohexane (1 : 1, 20 mL) by sonication in ultrasonic bath for 30 min. The mixture was filtered through glass wool containing anhydrous sodium sulfate for drying. The sodium sulfate was then washed with dichloromethane : cyclohexane (1 : 1, 5 mL). The extract was concentrated in rotary evaporator operated at 40°C and made up to 2 mL in cyclohexane [4].

The clean-up procedure for extracts was conducted according to Åkerblom (1995) with some modifications. A chromatographic tube of 10 mm i.d. × 32 cm was plugged with glass wool, packed with activated Florisil (3 g), and topped up with sodium sulfate (5–10 cm). The column was rinsed with cyclohexane (5 mL), and then the extract (2 mL) was passed through the column and eluted sequentially with cyclohexane (20 mL) and cyclohexane : acetone (9 : 1, 10 mL). The collected portions were combined and concentrated in rotary evaporator to 2 mL in cyclohexane : acetone (9 : 1).

### 2.3. Analytes Types

The analytes studied included fourteen (14) organochlorine compounds, three (3) organophosphorus pesticides, and one (1) pyrethroid pesticide. The organochlorine compounds were aldrin (1,2,3,4,10,10-hexachloro-1,4,4*α*,5,8,8*α*-hexahydro-exo-1,4-endo-5,8-dimethanonaphthalene), dieldrin (1,2,3,4,10,10-hexachloro-6,7-epoxy-1,4,4*α*,5,6,7,8,8*α*-octahydro-1,4-endo, exo-5,8-dimethanonaphthalene),* p,p*′*-*DDT (1,1,1-trichloro-2,2-bis (4-chlorophenyl) ethane),* o,p*′-DDT (1,1,1-trichloro-2-(2-chlorophenyl)-2-(4-chlorophenyl) ethane),* p,p*′*-*DDD (1,1-dichloro-2,2-bis (4-chlorophenyl) ethane),* o,p*′*-*DDD (1,1-dichloro-2-(2-chlorophenyl)-2-(4-chlorophenyl) ethane),* p,p*′-DDE (1,1-dichloro-2,2-bis (4-chlorophenyl) ethene),* o,p*′-DDE (1,1-dichloro-2-(2-chlorophenyl)-2-(4-chlorophenyl) ethene), *α*-endosulfan, *β*-endosulfan, *α*-HCH (*α*-1,2,3,4,5,6-hexachlorocyclohexane), *β*-HCH (*β*-1,2,3,4,5,6-hexachlorocyclohexane), *γ*-HCH (*γ*-1,2,3,4,5,6-hexachlorocyclohexane), and *δ*-HCH (*δ*-1,2,3,4,5,6-hexachlorocyclohexane). The organophosphorus pesticides were chlorpyrifos (*O,O*-diethyl* O*-3,5,6-trichloro-2-pyridylphosphorothioate), fenitrothion (*O,O*-dimethyl* O*-(3-methyl-4-nitrophenyl)-phosphorothioate), and pirimiphos methyl (*O*-(2-diethylamino-6-methylpyrimidin-4-yl)*O,O*-dimethyl phosphorothioate) and the pyrethroid pesticide was cypermethrin ((*RS*)-*α*-cyano-3-phenoxybenzyl(*1RS,3RS;1RS,3RS*)-3-(2,2-dichlorovinyl)-2,2-dimethylcyclopropane carboxylate).

### 2.4. Analytical Quality Assurance

All solvents and reagents were of analytical grade and above 99% purity (purchased from Thermo Fisher Scientific, UK). The glassware was cleaned with water and detergent and then with distilled water and rinsed with acetone. Other tools were also thoroughly cleaned before and after use. Sodium sulfate was heated at 130°C for 2 hours in order to remove moisture, and Florisil was preheated at 130°C overnight and partially deactivated with 5% distilled water. Pesticides standard solutions were of high purity (above 95%, obtained from Dr. Ehrenstorfer, Augusburg, Germany). Working standard solutions were prepared at concentrations ranging from 0.5 to 2 *μ*g/mL and were stored in a freezer. Blank and recovery tests were done to check the performance of the procedures and instruments. All sample types were analysed concurrently with matrix blanks; 6 blanks were analysed. A known volume of a mixture of pesticide standards solution was spiked into blank samples for recovery tests. Each spiked sample was homogenized, extracted, cleaned up, concentrated, and analysed just like the ordinary samples. Six recovery tests were done for the matrix blank samples. The detection limits of the analytes were established based on the lowest injected amounts in samples that resulted in peak heights three times higher than the baseline noise level. Every signal below this limit was treated as not detectable. No pesticides were detected in blank samples. The percentage recoveries for the analysed pesticides ranged from 71.2 to 110%. The recoveries were within the accepted range of 70–120% [5]. The detection limits of the analytes in samples ranged from 1.0 × 10^−4^ to 7.0 × 10^−4^ mg/kg.

### 2.5. Analysis, Identification, and Quantification

Sample analysis was done at Chemistry Department, University of Dar es Salaam. The compounds were determined using a gas chromatograph coupled to a mass spectrometer (GC-MS). The analyses were performed using a Shimadzu GC-MS QP 2010 Ultra equipped with MSD, fused silica capillary column Rtx-5MS (30 m × 0.25 mm × 0.25 *μ*m), and an autosampler. The initial temperature programme was 90°C, held for 2 min. The temperature was then ramped up to 260°C at 5°C/min and held for 5 min. The injector temperature was 250°C. The carrier gas was helium at the flow rate of 2.17 mL/min with average velocity of 54.6 cm/sec. The GC-MS was operated in splitless mode with a purge flow of 3 mL/min; the injection volume was 1 *μ*L and the pressure was set at 150 kPa. The GC interface temperature was 300°C. The mass spectrometer was operated in electron impact (EI) ionisation at 0.2 V with ion source temperature of 230°C and in full scan mode with the range of 45–500* m/z*. Standards were analysed on each day of analysis. Analysis was carried out in replicates (duplicates) and a total of 24 samples were analysed for each sample type (matrix).

**Figure 2:**
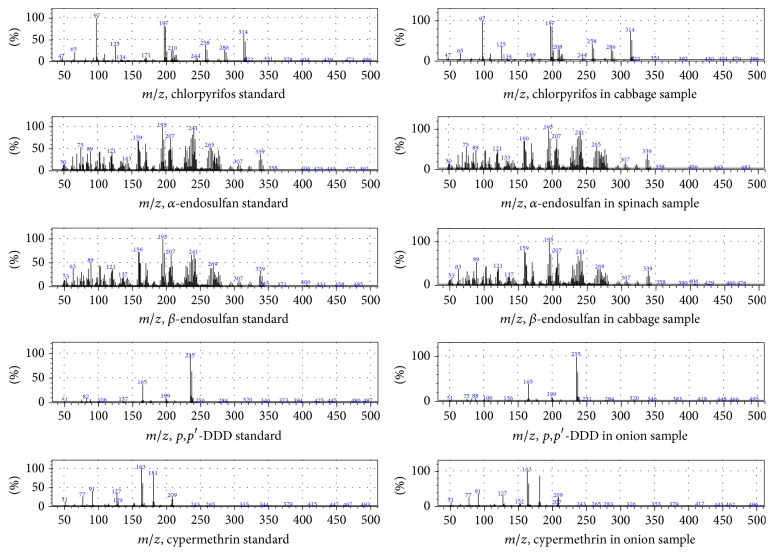
GC-MS full scan mass spectra of some analytes in standards and samples.

The identification of the compounds was accomplished by comparing the retention times and mass spectra of analytes in samples to those of reference standards run at the same conditions with the samples. The analytes were also identified using the NIST 11 mass spectral library (US National Institute of Standards and Technology). A specific pesticide was identified if it had the same retention time to that of the standard (within a deviation of ±0.05 min) and their spectra matched. Selected mass spectra of analytes are presented in Figure 2. Quantification was done by using peak heights. The mass fragment with the highest intensity was used for quantification while others were used as qualifying ions. The typical retention times and characteristic mass fragments (*m/z*) of analytes are given in Table 1.

### 2.6. Statistical Analysis

Statistical analysis of the data was performed by using GraphPad Instat software [6]. The data were subjected to Kruskal-Wallis test (Nonparametric ANOVA) to test for significance of variations followed by post-test (Dunn's multiple comparisons test).

## 3. Results and Discussion

### 3.1. Pesticide Residues in Cabbage Samples

Among the analysed pesticide residues, the following residues were detected in cabbage:* p,p*′*-*DDT,* p,p*′*-*DDD,* o,p*′*-*DDD, endosulfans, chlorpyrifos, and cypermethrin. Other pesticide residues analysed (*α*-HCH, *β*-HCH, *γ*-HCH, *δ*-HCH,* o,p*′-DDT,* o,p*′-DDE,* p,p*′-DDE, aldrin, dieldrin, fenitrothion, and pirimiphos methyl) were below the detection limits.  Table 2 presents the concentrations of detected pesticides and metabolites in cabbage samples.

#### 3.1.1. Organochlorine Pesticide Residues in Cabbage

Among the organochlorines detected in cabbage samples,* p,p*′*-*DDD was the most frequent pesticide residue although its concentrations in samples were generally low (up to (1.10 ± 0.20) × 10^−2^ mg/kg). Other DDT residues included* p,p*′*-*DDT which was detected in samples from one site with mean concentration of 4.00 × 10^−3^ mg/kg and* o,p*′*-*DDD was also detected in samples from one site with mean concentration of 1.00 × 10^−3^ mg/kg. The concentrations of total DDT (DDT + DDD + DDE) were up to 1.20 × 10^−2^ mg/kg. The DDD was found to appear in most samples (83.3%) while the DDE isomers were not detected. This suggested that the degradation of DDT was dominated by anaerobic pathway. DDD is a metabolite of DDT under anaerobic degradation, in which case irrigation water can create anaerobic environment [7]. The DDT residues found could be due to past use or environmental sources in the areas where the cabbages were grown.

The compounds *α*-endosulfan and *β*-endosulfan were detected in 33.3% of the samples and their concentrations were up to (6.00 ± 0.1) × 10^−1^ mg/kg and (2.10 ± 0.03) × 10^−1^ mg/kg, respectively. The concentrations of *α*-endosulfan were higher than *β*-endosulfan in all samples. This suggests recent inputs of fresh technical endosulfan or lack of significant degradation [8, 9]. The concentrations of endosulfans found in this study are generally lower than those found in cabbage samples from field areas by Meela [10] which were up to 2.0 mg/kg. The study conducted in India by Mukherjee [11] on pesticide residues in vegetables in and around Delhi showed that the concentrations of endosulfan in cabbage samples ranged from 0.15 to 0.59 mg/kg. These concentrations were comparable to those found in the present study.

#### 3.1.2. Organophosphorus and Pyrethroid Pesticides in Cabbage

Three types of organophosphorus pesticides (chlorpyrifos, fenitrothion, and pirimiphos methyl) were analysed in cabbage of which only chlorpyrifos was detected in 33.3% of the samples. The highest concentration of chlorpyrifos was 2.40 ± 0.04 mg/kg. The concentrations of chlorpyrifos were higher than other pesticide residues obtained. This can be explained by the fact that chlorpyrifos is a pesticide which is legalised for use in horticulture production [12]; thus it may represent the current use without significant degradation. Chlorpyrifos is a known human toxin. Once chlorpyrifos is released to the environment, it can travel through waterways and sediments and may enter the food supply by way of farming. It is used in farming extensively as a pesticide thereby exposing numerous food crops to its residues [13]. The only one pyrethroid analysed and detected in samples was cypermethrin. It was detected in 25% of the samples and the highest concentration was (3.00 ± 0.04) × 10^−2^ mg/kg, detected from Temeke samples. Cypermethrin is nonpersistent pesticide; it degrades easily in the presence of sunlight and water and this explains the detection of low levels in cabbage samples.

The findings of this study are comparable to the findings of the study conducted by Munawar and Hameed [14] on cabbage samples collected from markets in Pakistan that revealed the concentrations of chlorpyrifos ranging from ND to 0.075 mg/kg and those of cypermethrin ranging from ND to 0.14 mg/kg. The field study on cabbage samples conducted in Tanzania by Meela [10] did not detect the chlorpyrifos and cypermethrin; instead it reported fenitrothion at concentrations up to 2.56 mg/kg.

#### 3.1.3. Maximum Residue Limits (MRLs) Compliance

The concentrations of pesticide residues were compared to the maximum residue limits (MRLs) set by FAO and WHO [15], which refer to the upper legal levels of the concentrations for pesticide residues (expressed in mg/kg) in or on food or feed and the lowest possible consumer exposure to protect vulnerable consumers [16]. It is not expected to be exceeded in any food if the pesticide was applied in accordance with directions for its safe use. If a pesticide residue is found to exceed the MRL in a given foodstuff, the food commodity is said to be adulterated because it contains an illegal amount of the residue [15]. The concentrations of all DDD and all cypermethrin obtained in this study were significantly below their MRLs of 0.02 mg/kg and 1.0 mg/kg, respectively. The concentrations of endosulfans were less than the MRL in three samples and exceeded its MRL of 0.5 mg/kg by 1.6 times in one sample. All the concentrations of chlorpyrifos detected in samples significantly exceeded the MRL and the concentrations were 2–2.4 times greater than its MRL of 1.0 mg/kg. Generally, the concentrations of pesticide residues exceeded the MRLs in 41.7% of the cabbage samples. This implies that some farmers did not observe the withholding periods. Some of them spray the fields in the afternoon and pick the vegetables early in the morning for selling in the local markets. Violation of MRLs indicates threats to human health. Furthermore, there is a potential “cocktail effect” if humans are exposed to various pesticides, resulting in additive or synergistic effects. This means that even pesticides that were detected at “safe levels” may eventually pose health hazards to humans due to combined effects with other pesticides in the body [17].

### 3.2. Pesticide Residues in Spinach Samples

The following pesticide residues were detected in spinach:* p,p*′-DDD,* o,p*′-DDD, endosulfans, chlorpyrifos, and cypermethrin. Other pesticide residues analysed (*α*-HCH, *β*-HCH, *γ*-HCH, *δ*-HCH,* p,p*′-DDT,* o,p*′-DDT,* o,p*′-DDE,* p,p*′-DDE, aldrin, dieldrin, fenitrothion, and pirimiphos methyl) were not detected in all samples.  Table 3 presents the concentrations of detected pesticides and metabolites in spinach samples.

#### 3.2.1. Organochlorine Pesticides and Metabolites in Spinach

The most frequent organochlorine pesticide residue detected in spinach was* p,p*′-DDD with detection frequency of 75%, followed by endosulfan (33.3%) and* o,p*′*-*DDD (16.7%). The maximum concentration of* p,p*′*-*DDD was (6.40 ± 0.07) × 10^−1^ mg/kg. The highest concentration of total DDT was 6.50 × 10^−1^ mg/kg. The trend of DDT and its metabolites was the same as in cabbage samples. In Tanzania, the use of DDT has been prohibited for many years, but it is still in use for controlling malaria. On the other hand, DDT and its metabolites are persistent organic pollutants (POPs) and they remain in the environment for long periods; it is therefore not surprising to find residues of these pesticides in this study.

The highest concentrations of *α*-endosulfan and *β*-endosulfan in spinach were (2.40 ± 0.20) × 10^−1^ mg/kg and (8.00 ± 0.40) × 10^−2^ mg/kg, respectively. The concentrations of *α*-endosulfan were higher than *β*-endosulfan; this represents technical formulation, which suggests recent inputs or lack of significant degradation [8, 9]. Currently in Tanzania endosulfan is used as an insecticide registered under provisional registration category, sold under the trade name Thionex [12]. These concentrations are lower than the concentrations of endosulfans found in spinach samples collected from Sindh market in Pakistan, which were up to 1.28 mg/kg [18]. The study on* Amaranth* spp. from field areas in Tanzania reported concentrations of endosulfans of up to 1.69 mg/kg [10], which were higher than those found in this study.

#### 3.2.2. Organophosphorus and Pyrethroid Pesticides in Spinach

Chlorpyrifos was the only organophosphorus pesticide detected in 41.7% of the spinach samples. The highest concentration of chlorpyrifos was 3.00 ± 0.20 mg/kg. As in cabbage, the concentrations of chlorpyrifos were found to be higher than the concentrations of other pesticide residues obtained; this indicates the fresh use of chlorpyrifos with lack of significant degradation. Cypermethrin was detected in 33.3% of the spinach samples and the highest concentration was (4.00 ± 0.20) × 10^−2^ mg/kg.

The results obtained from this study show that the concentrations of chlorpyrifos were far above the concentrations obtained by Munawar and Hameed [14] who found that spinach samples collected from six markets in Pakistan contained chlorpyrifos at levels ranging from ND to 0.184 mg/kg while the concentrations of cypermethrin ranging from 0.01 to 0.156 mg/kg were comparable to the concentrations of cypermethrin obtained in this study. The field study on* Amaranths* spp. in Tanzania by Meela [10] did not detect chlorpyrifos and cypermethrin but reported fenitrothion at concentrations up to 0.243 mg/kg and fenvalerate at concentrations up to 4.123 mg/kg.

#### 3.2.3. MRL Compliance

Most of the concentrations of DDD residues in spinach were below the MRL while their concentrations in one sample were above the MRL of 0.2 mg/kg by 3.2 times. The concentrations of cypermethrin in all spinach samples were below the MRL. The concentrations of endosulfans and chlorpyrifos significantly exceeded the MRLs in all samples in which they were detected. The concentrations of endosulfan were 3.8–6.4 times greater than the MRL of 0.05 mg/kg, while those of chlorpyrifos were 2.6–6 times greater than the MRL of 0.5 mg/kg [15]. In general, the concentrations of pesticide residues exceeded the MRLs in 50% of the spinach samples. These findings suggest possible health hazards to the consumers. The level of contamination found could be linked to improper farmer practices; that is, the farmers may not be following good agricultural practices. It may be due to their ignorance about judicious use of pesticides [19].

### 3.3. Pesticide Residues in Onion Samples

Among the analysed pesticide residues,* p,p*′-DDD, *α*-endosulfan, *β*-endosulfan, chlorpyrifos, and cypermethrin were detected in 16.7–50% of the onion samples.  Table 4 shows their concentrations. Other pesticide residues analysed (*α*-HCH, *β*-HCH, *γ*-HCH, *δ*-HCH,* p,p*′-DDT,* o,p*′-DDT,* o,p*′*-*DDD,* o,p*′-DDE,* p,p*′*-*DDE, aldrin, dieldrin, fenitrothion, and pirimiphos methyl) were not detected.

#### 3.3.1. Organochlorine Pesticides and Metabolites in Onions

The organochlorine pesticide residues detected in onion samples were* p,p*′-DDD, *α*-endosulfan, and *β*-endosulfan. The concentrations of* p,p*′*-*DDD were very low, with maximum concentration of 1.02 × 10^−2^ mg/kg. Endosulfans were only detected in the samples collected from one market (Temeke). The highest concentrations of *α*-endosulfan and *β*-endosulfan were (2.20 ± 0.02) × 10^−1^ mg/kg and (7.00 ± 0.30) × 10^−2^ mg/kg, respectively. The concentrations of *α*-endosulfan were greater than those of *β*-endosulfan; this indicates the fresh use of technical endosulfan without significant degradation [8, 9]. The concentrations of endosulfans in this study are comparable to the concentrations obtained by Sheikh et al. [18] in onion samples collected from Sindh market in Pakistan which were up to 0.42 mg/kg.

#### 3.3.2. Organophosphorus and Pyrethroid Pesticides in Onions

Among the organophosphorus pesticide residues analysed in onion samples, only chlorpyrifos was detected in 25% of the samples and the highest concentration was 2.12 ± 0.10 mg/kg. Cypermethrin (pyrethroid) was detected in 16.7% of the samples and its highest concentration was (1.40 ± 0.04) × 10^−2^ mg/kg. The mean concentration of chlorpyrifos were higher than the levels obtained by Bempah et al. [20] in onion samples collected from Accra markets in Ghana, who revealed the mean concentration of 0.055 ± 0.011 mg/kg for chlorpyrifos.

#### 3.3.3. MRL Compliance

All the concentrations of DDD residues in onions were below their MRL of 0.2 mg/kg set by FAO and WHO [15]. The concentrations of total endosulfan exceeded the MRL of 0.05 mg/kg by 4.2–5.8 times. The concentrations of chlorpyrifos were 8.1–10.6 times above the MRL of 0.2 mg/kg. Cypermethrin was detected in two samples; the concentration of one sample was below the MRL of 0.01 mg/kg and one exceeded by 1.4 times. Generally, 33.3% of the onions had pesticide residues greater than the MRLs. The samples with residues above MRL may pose health hazards to the consumers. It may be due to lack of awareness of the farmers about the application dose, method of application, and withholding periods. The mismanagement or nonavailability of proper information about the pesticides application can lead to contamination of food crops with pesticide residues [19].

### 3.4. Variations of Pesticide Residues among Vegetables and Sampling Sites

There were variations in occurrence of DDT residues among the types of samples; they appeared most frequently in cabbage samples (83.3%) followed by spinach samples (75%) and onions (50%). In terms of occurrence of endosulfans, there were no variations in detection frequencies between spinach and cabbage samples (33.3%) while onions had the lowest detection frequency (16.7%). Chlorpyrifos most appeared in spinach samples (41.7%) followed by cabbage samples (33.3%) and finally onions (25%). The occurrence of cypermethrin varied in the order spinach > cabbage > onion samples, with detection frequencies of 33.3%, 25%, and 16.7%, respectively. Generally, 72.2% of the samples contained pesticides and metabolites. The compounds* o,p*′-DDT,* o,p*′-DDE,* p,p*′*-*DDE, aldrin, dieldrin, fenitrothion, pirimiphos methyl, and all of the HCH isomers (*α*-HCH, *β*-HCH, *γ*-HCH, and *δ*-HCH) were not detected in all samples. This suggested that they were not used for the vegetables studied or there was no significant contamination due to these compounds.

**Figure 3:**
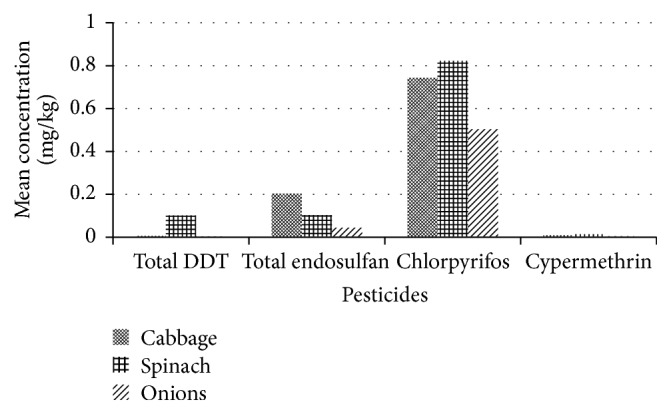
Mean concentrations of pesticide residues in samples.

There were slight variations in the concentrations of total DDT among all the sample types and the highest mean concentration was observed in spinach while the lowest was found in onion samples. There were slight variations in the levels of endosulfans; the highest mean concentration was found in cabbage samples and the lowest mean concentration was in onion samples. The highest mean concentration of chlorpyrifos was observed in spinach and the lowest in onion samples. For the levels of cypermethrin, spinach samples had the highest mean concentration while onion samples had the lowest. The concentrations of chlorpyrifos were the highest among the pesticide residues detected in the samples.  Figure 3 shows the variations in the mean concentrations of the residues.

Statistically, there were no significant variations in concentrations of the pesticide residues among the cabbage, spinach, and onion samples at 95% confidence level (Kruskal-Wallis statistics ranged from KW = 0.8206 at *p* = 0.6634 to KW = 5.065 at *p* = 0.0795; number of points = 12 in each sample type). Generally there were no significant variations of all pesticide residues among the vegetables. This suggests that there were no significant differences in the farming conditions as well as the application or contamination patterns of the pesticides and maybe the vegetables investigated have similar pesticides absorption or accumulation abilities.

There were no significant differences in the concentrations of pesticide residues among the sampling sites: KW = 3.256, *p* = 0.3538, and number of points = 21 in cabbage; KW = 1.433, *p* = 0.6979, and number of points = 18 in spinach, and KW = 2.067, *p* = 0.3558, and number of points = 15 in onions for most sites; except there were significant differences in the concentrations of residues in onions between Temeke and Mwananyamala samples (*p* < 0.05). These results indicate that most vegetables from all the markets have similar sources; they possibly come from similar places or regions.

## 4. Conclusions

Seven pesticide residues were detected, which were* p,p*′-DDT,* p,p*′-DDD,* o,p*′-DDD, *α*-endosulfan, *β*-endosulfan, chlorpyrifos, and cypermethrin. Their detection frequencies varied from 8.3% to 83.3%. The highest mean concentrations of total DDT, total endosulfan, and *α*-HCH were 6.50 × 10^−1^ mg/kg, 8.10 × 10^−1^ mg/kg, and 4.00 × 10^−3^ mg/kg, respectively. The mean concentrations of chlorpyrifos and cypermethrin were up to 3.00 mg/kg and 4.00 × 10^−2^ mg/kg, respectively. The profiles of DDT residues suggested past use dominated by anaerobic degradation forming* p,p*′-DDD. The concentrations of *α*-endosulfan were higher than the concentrations of *β*-endosulfan in all samples in which they were detected indicating contamination with fresh technical endosulfan. Residues of two or more pesticides were commonly found in the vegetables. The cooccurrence of pesticide residues in samples was due to various reasons including mixing of pesticides by farmers during application as farmers use more than one pesticide at one time due to resistance of pests. Other reasons could be due to contamination through water, soil, and air. There were no significant variations of the concentrations of pesticide residues among the vegetables suggesting similar contamination sources or patterns. The levels of pesticide residues were above the MRLs in 41.7% of cabbage samples, 50% of spinach samples, and 33.3% of onion samples. The samples could pose health hazards to the consumers. Therefore, it is recommended that effective monitoring of pesticide residues in food items is required.

## Figures and Tables

**Table 1:** List of pesticide standards/analytes with retention times and selected masses used as references in the GC-MS analysis.

Standards	Retention time	Quantification mass (*m/z*)	Qualifying ions (*m/z*)
*α*-HCH	17.38	181	51–145, 183–254
*β*-HCH	18.55	109	51–85, 145–254
*γ*-HCH	18.74	181	51–156, 183–254
*δ*-HCH	19.77	181	51–156, 183–254
Aldrin	22.83	66	79–293
*o,p*′-DDE	25.595	246	75–210, 248–318
*α*-Endosulfan	25.715	241	50–239, 243–339
Dieldrin	26.69	79	81–263
*p,p*′-DDE	26.78	246	75–210, 248–320
*o,p*′*-*DDD	27.07	235	88–199, 237, 239
*β*-Endosulfan	27.825	195	53–193, 197–339
*p,p*′-DDD	28.295	235	82–199, 237, 239
*o,p*′-DDT	28.38	235	75–199, 237, 239
*p,p*′-DDT	29.62	235	82–199, 237, 239
Fenitrothion	22.53	125	47, 79, 93, 109, 277
Chlorpyrifos	23.34	97	47, 65, 125–314
Pirimiphos methyl	22.68	290	56–276, 305
Cypermethrin	37.17	163	51–129, 165–209

**Table 2:** Mean concentrations (±SD) of pesticide residues in cabbage samples (mg/kg).

Site	Sample	*p,p*′-DDT	*p,p*′-DDD	*o,p*′-DDD	*α*-Endosulfan	*β*-Endosulfan	Chlorpyrifos	Cypermethrin
Kko	C1	ND	(9.00 ± 0.13) × 10^−3^	ND	(3.3 ± 0.01) × 10^−1^	(1.20 ± 0.002) × 10^−1^	2.40 ± 0.04	ND
	C2	ND	(4.00 ± 0.06) × 10^−3^	ND	ND	ND	ND	ND
	C3	ND	(1.10 ± 0.20) × 10^−2^	(1.00 ± 0.10) × 10^−3^	ND	ND	ND	ND

Bgn	C4	ND	ND	ND	ND	ND	ND	ND
	C5	ND	(1.00 ± 0.02) × 10^−3^	ND	ND	ND	ND	ND
	C6	ND	(3.00 ± 0.05) × 10^−3^	ND	ND	ND	2.30 ± 0.03	(3.00 ± 0.04) × 10^−2^

Mny	C7	ND	ND	ND	ND	ND	ND	ND
	C8	ND	(6.00 ± 0.10) × 10^−3^	ND	(2.50 ± 0.04) × 10^−1^	(9.00 ± 0.10) × 10^−2^	2.40 ± 0.01	(2.00 ± 0.03) × 10^−2^
	C9	(4.00 ± 0.10) × 10^−3^	(3.00 ± 0.04) × 10^−3^	ND	(6.00 ± 0.1) × 10^−1^	(2.10 ± 0.03) × 10^−1^	ND	ND

Tmk	C10	ND	(3.00 ± 0.10) × 10^−3^	ND	(2.80 ± 0.04) × 10^−1^	(9.00 ± 0.2) × 10^−2^	ND	ND
	C11	ND	(4.00 ± 0.17) × 10^−3^	ND	ND	ND	ND	ND
	C12	ND	(2.00 ± 0.01) × 10^−3^	ND	ND	ND	2.00 ± 0.03	(2.00 ± 0.02) × 10^−2^

Kko = Kariakoo; Bgn = Buguruni; Mny = Mwananyamala; Tmk = Temeke; SD = standard deviation of replicates; ND = not detected.

**Table 3:** Mean concentrations (±SD) of pesticide residues in spinach samples (mg/kg).

Site	Sample	*p,p*′-DDD	*o,p*′-DDD	*α*-Endosulfan	*β*-Endosulfan	Chlorpyrifos	Cypermethrin
Kko	S1	(1.00 ± 0.01) × 10^−3^	ND	ND	ND	ND	ND
	S2	(7.00 ± 0.10) × 10^−3^	ND	(2.10 ± 0.20) × 10^−1^	(7.00 ± 0.40) × 10^−2^	2.02 ± 0.10	(2.00 ± 0.10) × 10^−2^
	S3	ND	ND	ND	ND	ND	ND

Bgn	S4	(6.40 ± 0.07) × 10^−1^	(1.00 ± 0.01) × 10^−2^	ND	ND	ND	ND
	S5	(3.00 ± 0.02) × 10^−3^	ND	ND	ND	ND	ND
	S6	(4.00 ± 0.30) × 10^−3^	ND	(2.40 ± 0.20) × 10^−1^	(8.00 ± 0.40) × 10^−2^	3.00 ± 0.20	(4.00 ± 0.20) × 10^−2^

Mny	S7	(4.00 ± 0.10) × 10^−3^	(3.00 ± 0.20) × 10^−4^	(1.40 ± 0.01) × 10^−1^	(5.00 ± 0.30) × 10^−2^	1.31 ± 0.1	ND
	S8	(6.00 ± 0.20) × 10^−3^	ND	ND	ND	ND	ND
	S9	ND	ND	ND	ND	ND	ND

Tmk	S10	(1.10 ± 0.01) × 10^−2^	ND	ND	ND	2.10 ± 0.03	(2.00 ± 0.20) × 10^−2^
	S11	(2.00 ± 0.01) × 10^−2^	ND	(2.10 ± 0.10) × 10^−1^	(7.00 ± 0.06) × 10^−2^	1.60 ± 0.01	(1.00 ± 0.04) × 10^−2^
	S12	ND	ND	ND	ND	ND	ND

ND = not detected.

**Table 4:** Mean concentrations (±SD) of pesticide residues in onions (mg/kg).

Site	Sample	*p,p*′-DDD	*α*-Endosulfan	*β*-Endosulfan	Chlorpyrifos	Cypermethrin
Kko	O1	ND	ND	ND	ND	ND
	O2	(1.00 ± 0.01) × 10^−2^	ND	ND	ND	ND
	O3	(1.00 ± 0.02) × 10^−2^	ND	ND	1.84 ± 0.07	(6.00 ± 0.20) × 10^−3^

Bgn	O4	(1.00 ± 0.10) × 10^−3^	ND	ND	1.62 ± 0.06	(1.40 ± 0.04) × 10^−2^
	O5	(2.00 ± 0.10) × 10^−3^	ND	ND	ND	ND
	O6	ND	ND	ND	ND	ND

Mny	O7	ND	ND	ND	ND	ND
	O8	ND	ND	ND	ND	ND
	O9	ND	ND	ND	ND	ND

Tmk	O10	(1.00 ± 0.10) × 10^−3^	ND	ND	ND	ND
	O11	ND	(2.20 ± 0.02) × 10^−1^	(7.00 ± 0.30) × 10^−2^	ND	ND
	O12	(1.00 ± 0.10) × 10^−3^	(1.60 ± 0.01) × 10^−1^	(5.00 ± 0.20) × 10^−2^	2.12 ± 0.10	ND

ND: not detected.
